# Autophagy and the endolysosomal system in presynaptic function

**DOI:** 10.1007/s00018-020-03722-5

**Published:** 2020-12-19

**Authors:** Maria Andres-Alonso, Michael R. Kreutz, Anna Karpova

**Affiliations:** 1 Research Group Neuroplasticity, Leibniz Institute for Neurobiology, 39118 Magdeburg, Germany; 2 Leibniz Group ‘Dendritic Organelles and Synaptic Function’, Center for Molecular Neurobiology, ZMNH, University Medical Center Hamburg-Eppendorf, 20251 Hamburg, Germany; 3 Center for Behavioral Brain Sciences, Otto Von Guericke University, Magdeburg, Germany; 4 German Center for Neurodegenerative Diseases (DZNE), Magdeburg, Germany

**Keywords:** Autophagy, Endolysosomal system, Axonal boutons, Synaptic plasticity, Proteostasis

## Abstract

The complex morphology of neurons, the specific requirements of synaptic neurotransmission and the accompanying metabolic demands create a unique challenge for proteostasis. The main machineries for neuronal protein synthesis and degradation are localized in the soma, while synaptic junctions are found at vast distances from the cell body. Sophisticated mechanisms must, therefore, ensure efficient delivery of newly synthesized proteins and removal of faulty proteins. These requirements are exacerbated at presynaptic sites, where the demands for protein turnover are especially high due to synaptic vesicle release and recycling that induces protein damage in an intricate molecular machinery, and where replacement of material is hampered by the extreme length of the axon. In this review, we will discuss the contribution of the two major pathways in place, autophagy and the endolysosomal system, to presynaptic protein turnover and presynaptic function. Although clearly different in their biogenesis, both pathways are characterized by cargo collection and transport into distinct membrane-bound organelles that eventually fuse with lysosomes for cargo degradation. We summarize the available evidence with regard to their degradative function, their regulation by presynaptic machinery and the cargo for each pathway. Finally, we will discuss the interplay of both pathways in neurons and very recent findings that suggest non-canonical functions of degradative organelles in synaptic signalling and plasticity.

## Introduction

Neurons are highly polarized cells characterized by a complex dendritic arbour and an axon that emerges from the soma and bridges vast distances that can cover more than a meter in the human body. These neuronal processes are decorated by an enormous number of synapses that in principal neurons like pyramidal cells of the hippocampus can reach up to 15.000 synaptic contact sites [1, 2]. Synaptic neurotransmission comes along with huge metabolic demands [3, 4], elevated rates of protein turnover [5] and high membrane exchange that imposes significant challenges for the efficient delivery and constant supply of newly synthesized proteins. Likewise, removal of damaged proteins and organelles from synaptic sites and, in particular, from presynaptic boutons is essential to sustain synaptic function [6–8]. In addition, synapses are subject of activity-induced changes in their proteinaceous composition that underlie plastic processes in the context of learning and memory and that further exacerbate the complexity of local proteostasis [9–11]. Given the post-mitotic nature of neurons and their lifetime of several years and even decades, sophisticated mechanisms must be in place that assure the timely disposal of damaged proteins and organelles to ensure neuronal integrity [9, 12].

Several surveillance systems exist that monitor the physical and functional integrity of proteins and organelles and ensure the efficient removal of cargo [11, 13]. Degradation by these systems relies on the post-translational modification of targets—typically ubiquitination—that marks them for proteasomal or lysosomal degradation [14, 15]. Accordingly, degradation by the ubiquitin–proteasome system (UPS) has been intensively studied, synaptic cargoes of the UPS and tagging mechanisms have been identified, and its involvement in synaptic plasticity processes has also been described and reviewed elsewhere [14, 16, 17]. Lysosomes receive cargo for degradation from autophagy and the endolysosomal system, two distinct pathways characterized by the presence of different membrane-bound organelles of diverse origin that deliver cargo for degradation by ultimately fusing to lysosomes. Although these pathways have been extensively studied in non-polarized cells, their function in neurons and in particular at presynaptic sites is still far from being clear and many questions such as what is the cargo identity of each pathway at axon terminals, how local regulation occurs or how synaptic signalling itself affects protein removal still remain unanswered. In recent years, a significant effort has been made to better understand the role of these two pathways in axons and boutons and very recent studies unveil a potential role of these pathways in neurons beyond protein degradation. Here, we will revise these and other findings with regard to the function and regulation of these two degradative pathways at presynapses emphasizing their implications for synaptic function and plasticity.

## Autophagy in neurons

**Fig. 1:**
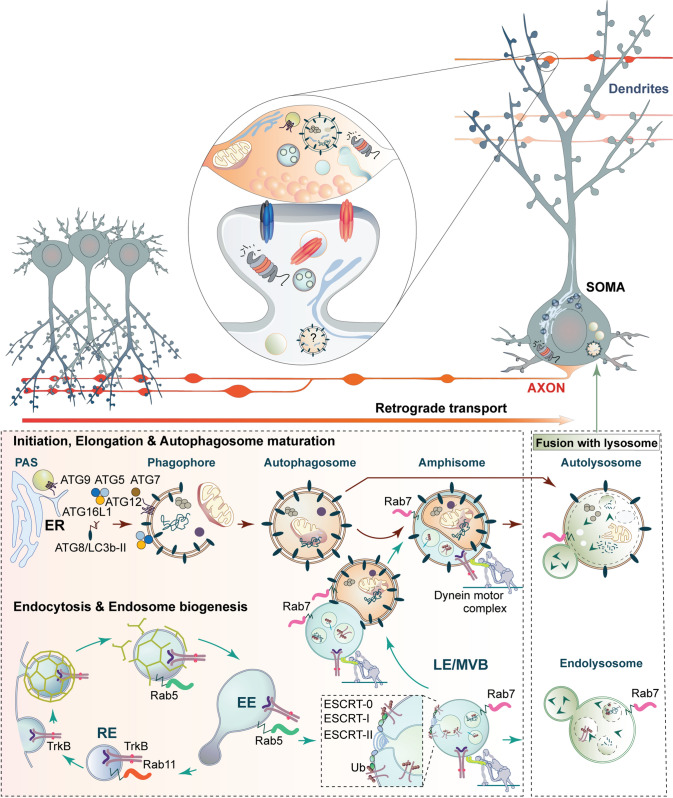
Overview of the two pathways that collect and deliver axonal cargo for degradation in lysosomes. Neurons pose a highly compartmentalized morphology endowed with a dendritic tree, the soma and a very long axon (in orange), and are decorated by many synaptic contacts (inset). Synapses are sites with high turnover rates and metabolic demands, where elements of autophagy, endolysosomal system and proteasomal-mediated degradation are present. Two main degradative pathways exist in axons that deliver cargo to lysosomes (described in the lower box) localized in the soma. In autophagy, autophagosome formation is initiated at distal axons at the phagophore assembly site (PAS). Cargo is sequestered by the phagophore which closes generating autophagosomes that are decorated by the autophagy adaptor LC3b-II. Autophagosomes are retrogradely transported in a dynein-dependent manner to the soma, where they fuse with degradative lysosomes generating autolysosomes. The endolysosomal pathway typically receives cargo from the plasma membrane by clathrin-mediated endocytosis and its fate is decided in early-endosomes (EE), organelles abundant in Rab5. Cargo is recycled back to the plasma membrane via recycling endosomes (RE) or is sent for degradation upon internalization into intraluminal vesicles by the ESCRT complex present in late endosomes (LE) and multivesicular bodies (MVB). EE matures into LE/MVB that are Rab7-positive. Cargo is then degraded in endolysosomes that originate from the fusion of LE/MVB with lysosomes. Amphisomes are generated upon the fusion of LE/MVB with autophagosomes and this is a necessary step for the acquisition of retrograde motors by autophagosomes. *Ub* ubiquitinated receptors

Macroautophagy (hereafter called autophagy) is a major degradative pathway for removal and recycling of cytosolic components, damaged organelles, misfolded and aggregated proteins. It is characterized by the sequestration of cargo through a cup-shaped, transient double-membraned structure called phagophore, whose membrane expands and ultimately fuses, generating a double-membrane vesicle called autophagosome that is endowed with an immense cargo capacity. Different membrane sources such as the endoplasmic reticulum (ER), ER-exit sites, the ER–Golgi intermediate compartment, the Golgi, the plasma membrane as well as recycling endosomes (RE) have been implicated to contribute to phagophore expansion [18, 19]. Following phagophore closure degradation of cargo is achieved by the fusion of autophagosomes to proteolytically active lysosomes. This process generates autolysosomes whose hydrolase-rich lumen eventually accomplishes cargo degradation (Fig. 1). The fusion of autophagosomes to late endosomes (LE) or multivesicular bodies (MVBs) results in an intermediate hybrid organelle of transient nature called amphisome that also fuses to lysosomes for cargo degradation (see Fig. 1).

Temporal and spatial control of autophagosome biogenesis and maturation is crucial to guarantee efficient cargo removal. This is achieved by a set of proteins encoded by the evolutionary conserved autophagy-related (*Atg*) genes that act in a tightly regulated, sequential manner to coordinate the different steps of autophagosome biogenesis up to fusion to the lysosome [18, 20]. Though initially considered as a bulk, non-selective process, it is nowadays believed that cargo is primed for degradation via direct association to so-called “autophagy receptors” that facilitate the recognition and degradation of specific cargo and that confer autophagy with cargo selectivity (reviewed in [19, 21]). Autophagy receptors associate to the lipidated form of the microtubule-associated protein 1 light chain 3b (LC3b-II) that is anchored to the inner and outer membrane of the phagophore, where it remains after closure of the autophagosome.

As compared to non-polarized cells, autophagy in neurons has adopted distinct features which likely arose as a result of demands imposed by the specific requirements of synaptic neurotransmission and neuronal morphology. First evidence arose from studies performed in transgenic mice in which either *Atg5* or *Atg7*, two genes that encode essential proteins in autophagosome biogenesis, were eliminated in neural precursor cells [22, 23]. Though the gross anatomy of the brain appeared normal in these animals, autophagy-deficient mice showed behavioural abnormalities and a closer histological examination revealed signs of neurodegeneration such as axonal swelling, accumulation of ubiquitinated proteins and increased neuronal cell death [22, 23]. Importantly, deficits in the function of the proteasomal system were not found in these mice, which indicates that effective clearance of cytosolic proteins by autophagy contributes to maintain neuronal integrity [23]. These initial studies also revealed a differential impact of the absence of ATG proteins depending upon on the brain region and the neuronal cell type. Purkinje cells of the cerebellum were the most vulnerable cell type in *Atg5* or *Atg7* knockout (ko) mice [22, 23] and selective deletion of *Atg7* in these cells caused an early, cell-autonomous axonal degeneration that preceded cell degeneration and locomotor deficits [24]. In contrast, deletion of *Atg7* in dopaminergic neurons [25] or ablation of Atg5 in excitatory neurons of the forebrain [26] did not induce neurodegeneration in young adult mice. Altogether, these studies indicate that autophagy plays an important role in maintaining neuronal homeostasis which in turn is essential to prevent neurodegeneration and behavioural deficits that will likely arise as a result of synaptic dysfunction and cellular loss.

The particular vulnerability of axons as compared to the somatodendritic compartment [24] was further confirmed in experiments performed in primary neurons. These studies revealed a high compartmentalization of the autophagy pathway in neurons, where autophagosome biogenesis is mainly found in distal axons and growth cones of developing and mature neurons [27, 28], whereas dendrites are predominantly devoid of these organelles ([27, 29] but see also [30, 31]), suggesting a predominant function in axons. Autophagosomes in distal axons contain ubiquitin-tagged cargo and organelles and are retrogradely transported in a microtubule-dependent manner to the soma, where degradative lysosomes are localized (Fig. 1) [27–29, 32]. Autophagosomes acidify towards proximal regions of the axon and at the same time they acquire late endosomal markers as a result of fusion events during retrograde transport (Fig. 1) [28, 33]. However, the fusion of autophagosomes with late endosomes might not only occur during transport but also at distal sites and this might be even a prerequisite for autophagosomes to acquire dynein motors [32].

Unlike in other cell types in which autophagy is induced upon starvation, autophagy in neurons is a constitutive process and starvation protocols involving nutrient deprivation have little or no effect on the rate of autophagosome formation [27, 29, 34]. In fact, the fundamental function of autophagy in controlling axonal homeostasis and/or eliminating cytoplasmic aggregation-prone proteins is essential to prevent the pathogenesis and progression of neurodegenerative diseases. This aspect has been extensively reviewed elsewhere [35–38].

When considering autophagy a fundamental cellular process, the late-onset neurodegeneration in mice lacking crucial *Atg* genes might be seen as a rather mild phenotype. In conjunction with the predominant localisation and function of autophagosomes in axons, this in turn suggests that autophagy might serve other and more specialized functions in neurons that are perhaps not restricted to proteostasis.

## The function of autophagy at axonal boutons

Biogenesis and presence of autophagosomes at presynaptic terminals has been extensively described in different neuronal cell types ranging from invertebrate organisms to mammals [25, 27, 39–41]. However, the underlying mechanisms as well as even simpler questions regarding the molecular identity of cargo, the existence of sensors for protein damage or the regulation of autophagy by synaptic activity are barely addressed.

Studies performed in dopaminergic neurons in which autophagosome biogenesis was prevented revealed that basal autophagy in this cell type contributes to the maintenance of presynaptic structure and function. Accordingly, neurons from 8-week-old transgenic animals lacking Atg7 expression in dopaminergic neurons presented an abnormal enlargement of their presynaptic terminals that were shown to release larger amounts of neurotransmitter and exhibited a faster presynaptic recovery [25]. These experiments were performed in neurons whose cell bodies had been severed, suggesting that the observed changes were mediated by the loss of local autophagy, and they revealed that basal autophagy acts as a repressor of presynaptic function in dopaminergic terminals [25]. In contrast, recordings from autaptic hippocampal cultures in which neurons grow isolated on astrocytic islands and establish synaptic contacts exclusively with themselves revealed no apparent effect of autophagy on neurotransmission. shRNA knockdown of ATG5 did not alter the amplitude of excitatory postsynaptic currents (EPSCs) as compared to control cells [42]. Interestingly, however, autophagy turned out to be essential for neurotransmission when selective protein damage was induced in axonal terminals by the generation of superoxides [42]. In this case the number of autophagic vacuoles was increased and ATG5 knockdown induced a significant reduction of EPSCs [42], suggesting that the removal of damaged proteins from terminals by autophagy is essential to maintain neurotransmission. These studies indicate that the significance of basal autophagy might differ at terminals from different neuronal cell types [22, 23]. This might be explained by diverse requirements for proteostasis based on different cytoarchitecture and metabolic demands. Axon terminals endowed with higher release rates are likely to be more sensitive to the lack of autophagy. Considering the predominant generation of autophagosomes at presynaptic boutons, the lack of evidence implying a function of basal autophagy in neurotransmission is stunning and more studies should be aimed to address this question. If the absence of autophagy has indeed no effect on basal neurotransmission this would indicate that cargo clearance from terminals by autophagy might only be important under conditions when demands for protein turnover are tremendously increased. Conceivable scenarios are high release rates due to burst firing and activity-driven changes in the molecular composition of boutons.

Although the role in the maintenance of basal synaptic transmission is still unclear, autophagy appears to play a crucial role in synapse development in invertebrates, though differences among cell types are also found here. While Drosophila larvae lacking different *Atg* genes show a significant reduction in the number of boutons at neuromuscular junctions (NMJs) [43], developing primary retinal neurons in the Drosophila brain show a significantly increased number of synapses in the absence of *Atg* genes [44]. Importantly, in the latter study authors report an intriguing mechanism by which autophagy controls synaptogenesis through regulation of the protein levels of two synaptic seeding factors, Liprin-α and Syd-1, that in turn affect filopodia stability and synapse formation in this cell type [44]. This study not only describes a function of synaptic autophagy in synaptogenesis but also identifies Liprin-α and Syd-1 as autophagy substrates. In vertebrates, studies of these two proteins suggest a role in synaptogenesis as well as synaptic vesicle (SV) docking and synaptic transmission [45, 46], but whether autophagy is involved in any of these remains unknown.

Finally, autophagy has also been implicated in regulating presynaptic assembly in a defined interneuron in *C. elegans.* In this study authors show that efficient delivery of ATG9, a transmembrane protein necessary for the formation of the phagophore, is crucial for autophagosome biogenesis and this appears to be necessary for the remodelling of axon terminals to enable proper SV clustering and localization of active zones [40].

### Local regulation of autophagy at presynaptic terminals

**Fig. 2:**
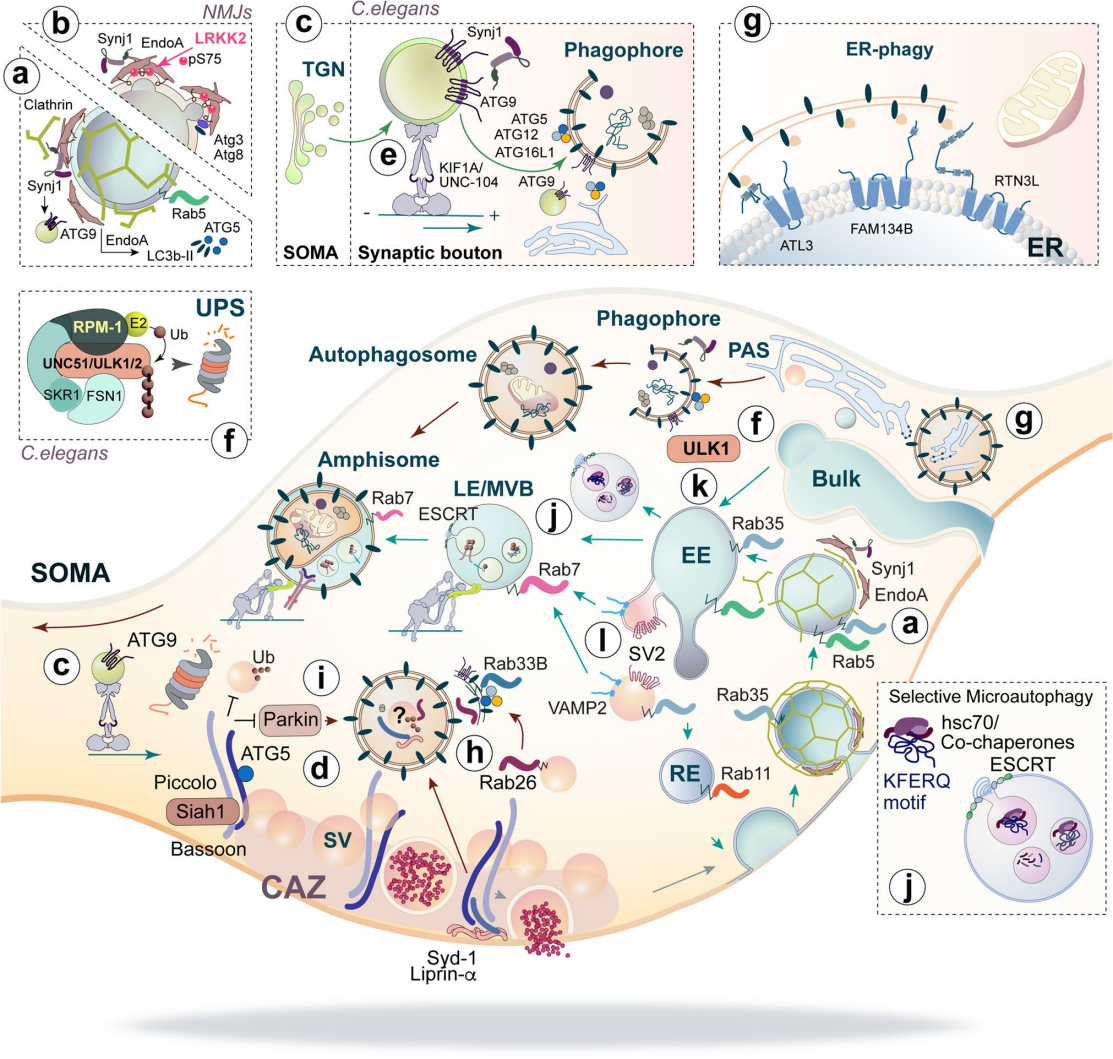
Molecular regulators and substrates of autophagy and the endolysosomal systems at presynaptic boutons. Presynaptic proteins regulate autophagy and autophagosome biogenesis at boutons. **a**, **b** EndoA and Synj1 mediate clathrin-mediated endocytosis and autophagosome formation. While phosphorylation of EndoA by LRKK2 regulates its insertion into and curvature of membranes (**a**), the phosphatase activity of the Synj1 SAC1 domain hydrolyses PIPs that are essential for autophagosome formation (**b**) and both EndoA and Synj1 might control the delivery of pre-autophagosomal membranes to the forming autophagosome via a direct interaction of Synj1 with ATG9 (**c**). **d** The active zone protein Bassoon represses local autophagy by sequestering ATG5, whereas autophagy turnover of another two presynaptic proteins, Liprin-α and Syd-1 controls synaptogenesis at Drosophila NMJs. **e**, **f** In *C. elegans*, autophagosome formation is regulated by the kinesin KIF1A/UNC-104 that mediates the anterograde transport of ATG9 (**e**), and the UPS, which controls the levels of the autophagy initiator UNC-51/ULK1/2 (**f**). **g**–**i** Presynaptic organelles are also substrates of autophagy. ER-phage takes place at boutons and several adaptors such as ATL3, FAM134B and RTN3L have been described (**g**). Degradation of SVs by autophagy is regulated by Rab26, which is involved in their delivery to autophagy membranes labelled by Rab33B (**h**), and Bassoon which mediates the ubiquitination of SV proteins via Siah and Parkin (**i**). Entry of integral proteins into the endolysosomal system occurs by clathrin-mediated or -independent endocytosis but proteins carrying a KFERQ motif might also enter this pathway by “endosomal microautophagy” (**j**). These proteins are recognized by the chaperone Hsc-70 which guides them to the endosomal membrane. Besides degradation by autophagy, SVs also undergo degradation via the endolysosomal system and their endosomal trafficking is regulated by Rab35 which has been shown to recruit ESCRT to SVs (**k**). SV2 and VAMP2 are degraded via this pathway, while other SV proteins like Synaptotagmin 1 are independent of this pathway (**l**)

Different types of stimuli have been shown to induce autophagy at presynaptic terminals such as treatment with the autophagy inducer Rapamycin [25, 42], synaptic activity [39, 47], or selective induction of synaptic protein damage [42], suggesting that the autophagy machinery can be engaged by different means. In addition, activation of different molecular pathways involving PKA [48] or G-protein coupled receptors [49], known to play important roles in neurotransmission, also regulate autophagy, indicating the existence of complex synaptic signalling networks that might control autophagy induction. Even though extensive work performed in invertebrates and non-polarized cells has revealed molecular mechanism involved in autophagosome biogenesis and maturation (reviewed in [20]), it is still unclear how such processes are regulated at spatially restricted compartments like presynaptic terminals that are subject to activity-dependent changes in protein composition and nanoscale organization. Notwithstanding, several independent studies have recently identified presynaptic proteins with well-established roles in presynaptic physiology as regulators of autophagy or autophagosome biogenesis at presynaptic boutons and will be discussed below (summarized in Fig. 2) [39, 40, 50–53].

Endophilin-A (EndoA) endocytic adaptors are key factors in clathrin-mediated endocytosis, an essential process for synaptic vesicle recycling (Fig. 2a). Initial studies performed in mice lacking the three isoforms of EndoA suggested a role of these adaptors in regulating autophagosome biogenesis, as evidenced by the decreased levels of the autophagy proteins LC3b-II and ATG5 in triple ko mouse brain and the low number of autophagosomes detected in primary neuronal cultures prepared from those animals [51]. This effect is independent of the role of EndoA in endocytosis since levels of ATG16L1 and ATG9, two essential autophagy factors that undergo endocytosis in a clathrin-dependent manner [54] were unaffected [51]. The molecular mechanism by which EndoA regulates autophagosome formation was subsequently deciphered at Drosophila NMJs [39], where it was shown that the phosphorylation of the amphipathic helix H1 of EndoA changes the ability of the protein to insert in membranes. While non-phosphorylated EndoA inserts deeply in the lipid bilayer and causes a shallow membrane curvature, phosphorylation mediated by LRKK2 induces the protein to insert less deep. This triggers pronounced membrane curvatures to which autophagic factors such as Atg3, a protein involved in LC3 lipidation, are recruited (Fig. 2b) [39]. Therefore, phosphorylation of EndoA by LRKK2 serves as a molecular switch to induce autophagy at NMJs, where EndoA is essential for autophagy in vivo [39]. Since LRKK2 is one of the most commonly mutated proteins in Parkinson's Disease [55], these results might be of particular relevance in the context of this neurodegenerative disease.

Interestingly, EndoA also interacts with Synaptojanin1 (Synj1) (Fig. 2a–c), a lipid phosphatase enriched in synapses [53]. Synj1 localizes to pre-autophagic membranes containing ATG9 that are delivered to the forming autophagosome. This protein contains two phosphatase domains and the second one, termed SAC1, hydrolyses phosphoinositide phosphates (PIP) such as PI(3)P and PI(3,5)P2, which are recognized by different autophagy proteins that promote autophagosome formation. Interestingly, mutations within the SAC1 domain are also found in patients of Parkinson’s Disease [56]. In this study, Drosophila larvae lacking Synj1 or expressing a phosphatase-deficient mutant showed defective induction of autophagy at NMJs [53] as well as an accumulation of cisternae resembling early autophagic intermediates at terminals, suggesting that PI(3)P and PI(3,5)P2 dephosphorylation by Synj1 is essential for autophagosome maturation at synapses [53]. Because Synj1 interacts with EndoA, these two proteins are likely to act in concert to control early-autophagosome biogenesis in a process that involves the interaction of Synj1 with ATG9 and the delivery of pre-autophagosomal membranes to the forming autophagosome (Fig. 2c) [53].

Two core scaffold proteins of the presynaptic active zone, Piccolo and Bassoon, have also a function in regulation of autophagy at boutons. This was demonstrated in a recent study in which shRNA-based downregulation of Piccolo and Bassoon protein levels triggered the accumulation of autophagic vacuoles at synaptic terminals as well as periaxonal regions, which suggested a functional retrograde transport of these vacuoles [27, 29]. Interestingly, loss of Bassoon turned out to be sufficient to induce autophagy at synaptic terminals and overexpression of the full-length protein had the opposite effect, indicating that the presence of Bassoon locally regulates autophagy at boutons [52]. This regulation takes place via a direct interaction between the coiled-coil 2 domain of Bassoon and the LC3b-II ligase ATG5, which sequesters ATG5 at terminals and prevents autophagosome formation (Fig. 2d) [52]. Of note, Bassoon has recently been shown to also inhibit proteasomal activity within the presynaptic compartment by a directly interaction with a subunit of the 20S core proteasome [57].

Two alternative mechanisms of local autophagy regulation that are not mediated by proteins with an exclusive role at presynapses have been described in *C. elegans*. The first one relies in the function of the kinesin UNC104/KIF1A, which participates in autophagosome formation by enabling the delivery of ATG9-enriched vesicles in an anterograde manner to the terminals of a specific interneuron (Fig. 2e) [40]. As mentioned above, ATG9 is a transmembrane protein localized in small vesicles that are recruited to sites of phagophore formation. In agreement with a lack of pre-autophagosomal membranes, the absence of UNC104/KIF1A induced a significant reduction in autophagosomes at synapses even in the presence of all essential ATG proteins, indicating the fundamental roles of this kinesin and, in general, anterograde transport for autophagosome formation [40]. The second mechanism of regulation of autophagy induction depends on the activity of the UPS and RPM1, an atypical RING E3 ubiquitin ligase that degrades UNC-51 in specific axonal compartments via its ubiquitin ligase activity (Fig. 2f). UNC-51 is the *C.elegans* orthologue of the mammalian ULK1/2, a primary initiator of autophagy, whose degradation constrains autophagy induction. Accordingly, *rpm-1* mutants showed an increased number of axonal autophagosomes and a phenotype with regard to axonal termination and synapse maintenance that was abolished in the absence of other autophagy genes such as *atg9*, suggesting a causal role of autophagy [58]. In this interplay between proteasomal degradation and autophagy the degradative activity of the proteasome serves to restrain autophagy induction at axon terminals by degrading key players of the autophagy machinery.

### Substrates of autophagy at boutons

Specific removal of cytosolic components and organelles by autophagosomes takes place through cargo receptor proteins that associate to cargo and the membrane-bound cargo adaptor LC3b-II, thereby tethering cargo to nascent autophagosomes. Cargo adaptors for the selective removal of different organelles including among others mitochondria, lysosomes, peroxisomes and the nucleus have been identified in different organisms (reviewed in [19]). Despite compelling evidence for autophagosome formation at presynaptic sites [59] and a supportive role of autophagy in axonal and synaptic proteostasis, little is known about the molecular identity of cargoes and cargo adaptors of selective autophagy at synapses as well as its function and regulation by synaptic activity.

Mitochondria play a crucial role in supporting synaptic communication by means of energy supply and calcium buffering at synapses. Selective removal of these organelles by autophagy, a process termed mitophagy, has been shown to occur at distal axons [59] and mitochondria are commonly found in autophagosomes [60]. Likewise, the molecular mechanisms of mitophagy are well-studied [61, 62] and mutations in the key mitophagy adaptors, PINK1 and Parkin, are linked to early onset familiar Parkinson's Disease [63]. Another organelle tightly linked to autophagy is the ER, a continuous network of tubuli that extends throughout the neuronal cytoplasm and that participates in the synthesis of essential cellular components such as membrane, lipids and transmembrane proteins as well as calcium storage. In axons, smooth ER tubuli branch at terminals forming a network of cisternae that surrounds synaptic vesicles and other organelles such as endosomes [64]. Autophagy is closely related to the ER, since autophagosome formation takes place on or in very close proximity to tubuli and the ER has been also postulated to be one of the major compartments that supply autophagosomes with membranes (Fig. 2g) [65]. Recently, several cargo adaptors have been described to mediate ER-phagy, the selective degradation of ER by autophagy (reviewed in [65]). Some of these adaptors such as FAM134B, ATL3 or RTN3L (see Fig. 2g) are highly expressed in the brain, and point mutations in FAM134B or ATL3 are associated to hereditary sensory and autonomic neuropathies though mechanisms are still poorly understood [66, 67]. Importantly, ER-phagy regulates the ER size and its remodelling via FAM134B, which plays a role in the coordination of ER scission and subsequent sequestration of ER by autophagosomes that is essential for cell survival [68]. Since the function of the ER in buffering and releasing calcium directly impacts neurotransmitter release [69], changes in its morphology could directly impact presynaptic function and have implications in presynaptic plasticity. Interestingly, a recent study in non-polarized cells that pursued a genome-wide ER-phagy screen unmasked two unexpected pathways required for ER-phagy: mitochondrial metabolism and ER-localized UFMylation [70]. ER-phagy does share effectors with general autophagy but while inducers of ER-stress do not lead to ER-phagy, inhibition of ER-phagy does result in ER stress and the unfolded protein response, suggesting an essential role of ER-phagy for maintaining ER integrity [70]. Moreover, an extensive interplay exists between mitochondria and ER-phagy in which functional mitochondrial metabolism was found to be important for ER-phagy. The second pathway necessary for ER-phagy is UFMylation, a ubiquitin-like post-translational modification that favours protein stability. UFMylation of ER surface-localized substrates is necessary for ER-phagy and some of the identified substrates include ribosomal components and a glycosylation enzyme [70]. Because glycans serve quality control, this opens up the possibility that ER-phagy might contribute a mechanism of surveillance for nascent proteins at synapses, a process that could serve important functions to maintain the integrity of presynaptic machinery.

SVs undergo several rounds of recycling at boutons posing a challenge for membrane sorting and a high metabolic demand. Surprisingly, how degradation of SVs and SV proteins takes place and what is the underlying predominant mechanism is currently unclear (see also “Molecular organizers and cargo of the endolysosomal system at presynaptic sites”). A few studies have identified SVs as substrates of the autophagy pathway [25, 50, 71, 72] and recent evidence describes the involvement of Rab26-dependent pathways in mediating the delivery of SVs to pre-autophagosomal membranes [50, 72]. Accordingly, Rab26 associates to SVs and is particularly enriched in large membrane-surrounded clusters that are also positive for proteins of the early autophagy machinery such as ATG16L1, Rab33B and LC3b-II and whose formation correlates with high levels of Rab26 [50], suggesting that this protein acts as an intermediate between SVs and the autophagy machinery (Fig. 2h). Rab26 is a small GTPase protein whose activation relies on a group of proteins called GTPase Activator Proteins (GEFs). Motorneurons from transgenic mice lacking the GEF Plekhg5 and in which Rab26 activation is deficient show accumulation of SV proteins and enlarged SVs that result in motorneuron disease [72], indicating that both Rab26 and Plekhg5 participate in the autophagy-dependent degradation of SVs. Another protein involved in the turnover of SVs in autophagosomes is the active zone protein Bassoon, a protein that also represses presynaptic autophagy via its interaction with ATG5 (see above; Fig. 2d) [52]. In a recent study it was reported that Bassoon mediates the ubiquitination of SV proteins via two E3-ubiquitin ligases, Siah1 and Parkin, which in turn drives SV proteins for autophagy degradation (Fig. 2i) [71]. Accordingly, absence of Bassoon causes an increase of ubiquitinated SV proteins that leads to a decrease of SV protein levels in an autophagy-dependent manner, and this correlates with a smaller SV pool size and an overall higher rate of SV protein turnover found in Bassoon KO neurons [71].

These studies provide evidence for the involvement of autophagy in the turnover of SVs, and presence of SVs in autophagosomes has been indeed reported at EM level (see for example [71]). However, degradation of SVs and SV proteins via the endolysosomal system was also reported (see “Molecular organizers and cargo of the endolysosomal system at presynaptic sites”) and the vast majority of synaptic autophagosomes are devoid of SVs. Therefore, alternative pathways might exist for the turnover of these organelles, especially taking into consideration the high abundance of SVs at single terminals and their elevated turnover rates. In line with this, studies performed to address lifetimes of SV proteins revealed high heterogeneity, arguing against the elimination of SVs as discrete units [7] and rather support a model in which SV proteins are sorted individually by the machinery of the endolysosomal system (see below). Protein sorting in the endolysosomal system might be an efficient means to remove damaged proteins, while degradation via autophagy might be triggered upon specific conditions that require a fast, bulk degradation of discrete SVs.

### Autophagy in dendrites

Evidence for the presence of autophagosomes at postsynapses and in dendrites is sparse. However, some reports have suggested a role of autophagy in regulating postsynaptic structure and function. Dopaminergic and noradrenergic neurons lacking *Atg7* exhibit dystrophic dendritic trees containing large inclusions that lacked a limiting membrane [73]. Apart from this, ultrastructural analysis of synapses did not reveal major alterations suggesting that, unlike in dopaminergic axons, absence of autophagy did not significantly affect postsynaptic morphology [25, 73]. Nevertheless, an association between autophagy in the dendritic compartment and neuropsychiatric diseases has been described in the context of developmental spine pruning [74, 75]. During development, excessive synapse formation takes places in the mammalian cortex that is counteracted by subsequence synapse elimination at later stages. In brains from autism spectrum disorder (ASD) patients an excess of dendritic spines was reported that is associated with a reduction of autophagy levels [74]. It was speculated that this reduction in autophagy levels was induced by a hyperactivation of the autophagy repressor mTOR which might cause inhibition of autophagy and a deficit in pruning that, in turn, might at least in part be responsible for the abnormally high synapse density found in ASD brains [74]. Interestingly, deficits in spine pruning are also observed in the cortex of conditional *Atg7* mice and hippocampal primary neurons of fragile X mice, a heritable form of intellectual disability which is also associated to decrease autophagy levels due to mTOR dysregulation and to aberrant spine density [75]. Levels of two postsynaptic proteins, PSD95 and Arc, are increased and their expression is normalized by activation of autophagy, suggesting that both proteins could be degraded by the autophagy pathway [75]. Besides unmasking an association between autophagy and neuropsychiatric diseases, these studies also reveal a role of autophagy in the assembly and maturation of neural circuits. This function does not seem to be restricted to neuronal cells, since transgenic mice in which autophagy has been ablated from microglial cells, known to participate in developmental synapse pruning, also present altered synapse number and brain connectivity [76].

Interestingly, PSD95 and two other postsynaptic density scaffolding proteins, PICK1 and Shank3, have also been shown to associate to the autophagosome marker LC3b-II in hippocampal lysates, suggesting that these proteins could constitute substrates of the autophagy pathway [77]. In this study, an increased number of autophagosomes in axons, dendritic profiles and spines was reported in neurons from a transgenic mouse line in which *bdnf* was eliminated. Although independent of developmental pruning, downregulation of autophagy by BDNF also correlated with an enhanced spine density and higher levels of PSD-95 [77], altogether suggesting that autophagy in dendrites could regulate spine density.

Turnover of AMPA receptors, a key protein in postsynaptic function that plays an essential role in neurotransmission and synaptic plasticity has also been described to be at least in part mediated by autophagy [78]. Bath application of NMDA aimed to induce chemical long-term synaptic depression resulted in an elevation of LC3b-II levels that correlated with a decrease in total levels of the AMPA receptor subunit GluA1 [78].

Given the sparse presence of autophagosomes in dendrites, how does autophagy impact synaptic pruning and postsynaptic function? A very recent study suggests the induction of local autophagosome biogenesis in dendrites upon induction of long-term synaptic depression (LTD)—a form of synaptic plasticity characterized by the shrinkage and elimination of dendritic spines [30]. These autophagosomes, when biochemically purified, seem to contain postsynaptic cargo such as scaffold proteins and cell-adhesion molecules [30]. Thus, unlike the constitutive and basal autophagosome biogenesis in axons, autophagy in dendrites could be induced “on-demand” in the context of long-term plasticity processes. Surprisingly, another recent report has obtained opposite results as they found that inhibition of dendritic autophagy is essential for the induction of LTD [31], since autophagy impairs the LTD-mediated internalization of AMPA receptors [31]. However, since Rapamycin was used to induce autophagy, it will be important to determine whether induction of autophagy by endogenous mechanisms such as synaptic stimulation itself triggers similar effects [31].

## Endolysosomal degradation in neurons

Degradation of cargo deriving from the plasma membrane such as integral membrane proteins and lipids is typically mediated by their entry into the endolysosomal system, a complex network of membrane organelles from where cargo will be either recycled or delivered to lysosomes for degradation (see Figs. 1, 2). Transmembrane proteins and their associated factors such as receptor ligands and lipids generally access this pathway by clathrin-mediated endocytosis [79], though clathrin-independent forms of endocytosis such as pino- and phagocytosis have also been reported [80]. Following internalization, several cargo-containing transport vesicles undergo homotypic fusion or fuse to an already existing endosome, a so-called “early-endosome” (EE), which constitutes the first compartment receiving cargo from the plasma membrane [81]. At this level, cargo tagged for degradation by ubiquitination is detected by the endosomal sorting complexes required for transport (ESCRT) which retrieves it to endosomal subdomains and facilitates the formation of intraluminal vesicles (ILVs) that will be subsequently delivered to lysosomes for cargo degradation (Figs. 1, 2) [82]. Cargo sorting takes places in EE but also in late endosomes (LE) which result from the maturation of EE, a process characterized by an increased luminal acidification and the switch of Rab proteins that differentially localize along distinct compartments, with Rab5 being abundant in EE and Rab7 mainly in LE [83]. This so-called “Rab conversion” is essential for the long-range transport of late endosomes in axon and dendrites towards the soma [84–86], where ILVs will be degraded upon fusion with catalytically active lysosomes (Fig. 1) [83].

Cytosolic proteins might also enter the endolysosomal system by a process called “endosomal microautophagy” (Fig. 2j). This process, independent on the canonical autophagy machinery, is selective for degradation of proteins harbouring a KFERQ sequence recognized by the cytosolic chaperone Hsc70 which guides cargo proteins to the endosomal membrane [87].

Like observed in autophagy-deficient neurons, proper functioning of the endolysosomal pathway and, in particular, of the endomembrane system and its molecular organizers, is essential for neuronal function and defects in membrane trafficking are hallmarks for neurodegeneration (for review, see [88, 89]).

### Molecular organizers and cargo of the endolysosomal system at presynaptic sites

As outlined above some reports suggest the involvement of autophagy in SV turnover [25, 50, 71, 72]. However, studies in Drosophila NMJs revealed that following endocytosis SV localize in endosomal compartments labelled with EE markers [90]. This observation was made when studying mutants of Skywalker, a GTPase-activating protein (GAP) for Rab35, a Rab protein localized in the plasma membrane that is involved in endosomal trafficking and clathrin-dependent endocytosis. In Skywalker mutants in which Rab35 is overactivated, synaptic stimulation led to an accumulation of cisternae-like structures at the expense of SVs (Fig. 2k). Ultrastructural analysis of these cisternae combined with dye-based labelling of SV membranes revealed that these membranes localize to endosomal compartments following endocytosis upon activity-induced SV recycling [90]. Rab35-dependent membrane trafficking via endosomal compartments provoked a more efficient refilling of the readily-releasable SV pool and correlated with an enhanced rate of neurotransmitter release, effects that were depending on the function of the ESCRT complex involved in sorting of damaged proteins for degradation [90]. Altogether, this study provides evidence for the trafficking and sorting of SV membrane components at endosomal compartments, a process that is regulated by Skywalker/Rab35/ESCRT and that has a direct impact on neurotransmitter release [90]. In hippocampal primary neurons, Rab35/ESCRT was also shown to mediate the degradation of a subset of SV proteins, SV2 and VAMP2, upon synaptic stimulation [91]. In this study, activity-dependent activation of Rab35 seemed to promote the turnover of SV2 and VAMP2 by directly interacting with Hrs, the initial component of ESCRT that binds and recruits ubiquitinated substrates (Fig. 2l). Interestingly, turnover via this mechanism is exclusive for some proteins as levels of Synaptotagmin1, another SV protein, remained unaffected [91] suggesting selective degradation of SV proteins and arguing against the bulk degradation of single SVs as expected for autophagy-mediated turnover. Interestingly, in this study the EE-associated protein Rab5 was shown to be implicated in the degradation of not only SV2 and VAMP2 but also active-zone proteins such as SNAP25 and Munc13 [91]. Unlike Rab35, Rab5 does not interact with Hrs and Rab5-mediated degradation may be accomplished by alternative pathways. In line with this, a role of Rab5 in the early steps of the autophagy pathway has been described [92] and it is tempting to hypothesize that while playing an essential role in sorting of proteins at the EE level, Rab5 could also regulate the degradation of defined targets by activating autophagy [92]. Finally, the motor adaptor Snapin that controls dynein-dependent retrograde transport of vesicles from the endolysosomal pathway to the soma has also been shown to regulate the turnover of SVs and to impact the size of presynaptic SV pools [93]. In line with this, neurons lacking Snapin present enlarged presynaptic terminals that accumulate SV markers as well as degradative structures, in agreement with an impaired retrograde transport of both organelles. Impairment of this transport directly affected the SV pool size at terminals, suggesting that SV components are trafficked in a Snapin-dependent manner along the endolysosomal route to the soma for degradation [93]. Importantly, accumulated autophagosomes found in boutons of Snapin-deficient neurons did not contain SVs in their lumen, further suggesting that autophagy is not the predominant pathway of SV turnover [93]. Altogether, these studies evidence the complexity of the existing mechanisms that ensure the turnover of single proteins at presynaptic terminals with high specificity. Also they manifest the necessity of further investigations to better understand how cargo sorting and selectivity is achieved at the molecular level.

Studies performed in vivo in Drosophila brains have shed light into mechanisms of cargo-specific endolysosomal sorting and degradation at presynaptic terminals. Making use of cargo proteins tagged with fluorophores that allowed the discrimination of different vesicular compartments, authors studied cargo sorting and degradation at terminals and found that two distinct endolysosomal “hubs” exist that exclusively receive cargo from either SVs or the plasma membrane [94]. These two hubs are endowed with different molecular identity: while maturation of the SV hub depends on the delivery of the Cathepsin-l-like protease CP1 by the *N*-ethylmaleimide-sensitive factor attachment protein receptor (SNARE) Synaptobrevin (n-Syb) and acidification by the vATPase component v100, maturation of the plasma membrane hub is depending on Rab7. This study demonstrates the existence of at least two molecularly distinct pathways that sort proteins locally at terminals for degradation and further underscores the complexity of sorting and degradation mechanisms in place to ensure the efficient selective removal of proteins at the presynaptic terminal. Furthermore, the presence of CP1 in the SV hub described in this study suggests the possibility that SVs might be locally degraded at axon terminals in these neurons [88].

Endosomal microautophagy has also been shown to participate in the turnover of a subset of presynaptic proteins in *Drosophila* [95]. This is controlled by Hsc70-4, a chaperone that besides its chaperone activity, mediates endosomal membrane deformation and microautophagy in synapses (Fig. 2j). Activity of Hsc70-4 selectively regulates the turnover of some synaptic proteins like Unc-13/Munc13 and EndoA among others that harbour microautophagy recognition motifs [95] but it does not regulate the turnover of all proteins carrying recognition motifs, highlighting again the high specificity of different degradation pathways for the turnover of single proteins. Importantly, membrane deformation mediated by Hsc70-4 increase the ready-releasable pool of SV and promotes neurotransmitter release, suggesting that endosomal microautophagy, by efficiently removing damaged, older proteins, facilitates synaptic transmission [95]. Since microautophagy recognition motifs are abundant among synaptic proteins and Hsc70 and other co-chaperones are also present in vertebrate synapses, this pathway could provide an efficient means to selectively and efficiently remove single proteins from this compartment.

Finally, as described for autophagy two proteins have been identified that, besides their known primary function, also play a role in the degradation of cargo by the endolysosomal system [41, 96]. The first one is the vesicular ATPase (vATPase), a multi-subunit complex with a role in acidification of intracellular compartment and membrane protein sorting and degradation. The neuron-specific *v100* gene encodes the α1 subunit of the V0 sector of vATPase that is well-known to play a role in the acidification of endosomes and their maturation into degradative compartments. Besides this function, V100 has been shown in Drosophila to enable the fusion of cargo-containing carriers with EEs in an acidification-independent manner [41], thereby mediating the entry of cargo into the endosomal pathway. Drosophila mutants carrying an acidification-deficient form of the protein accumulate enlarged degradation-incompetent vesicles that likely arise as a consequence of the efficient fusion of EE into larger compartment that are unable to mature, and this correlates with a neurodegenerative phenotype [41]. The second known protein with a function in cargo degradation by the endolysosomal system is neuronal Syb, is a vesicle SNARE that besides playing a role in SV exocytosis also mediate the delivery of vesicles containing degradative machinery such as proteases and V100-containing vATPases to endosomes. As observed in *v100* mutants, *n-Syb* mutants show accumulation of small vesicles and large degradative compartments that seems to arise from a defect in vesicle fusion and that ultimately lead to neurodegeneration in Drosophila [96]. Together with V100, n-Syb plays a role in a “sort-and-degrade” pathway that fine-tunes the degradative capacity of the neurons.

## Amphisomes: when late endosomes meet autophagosomes

**Fig. 3:**
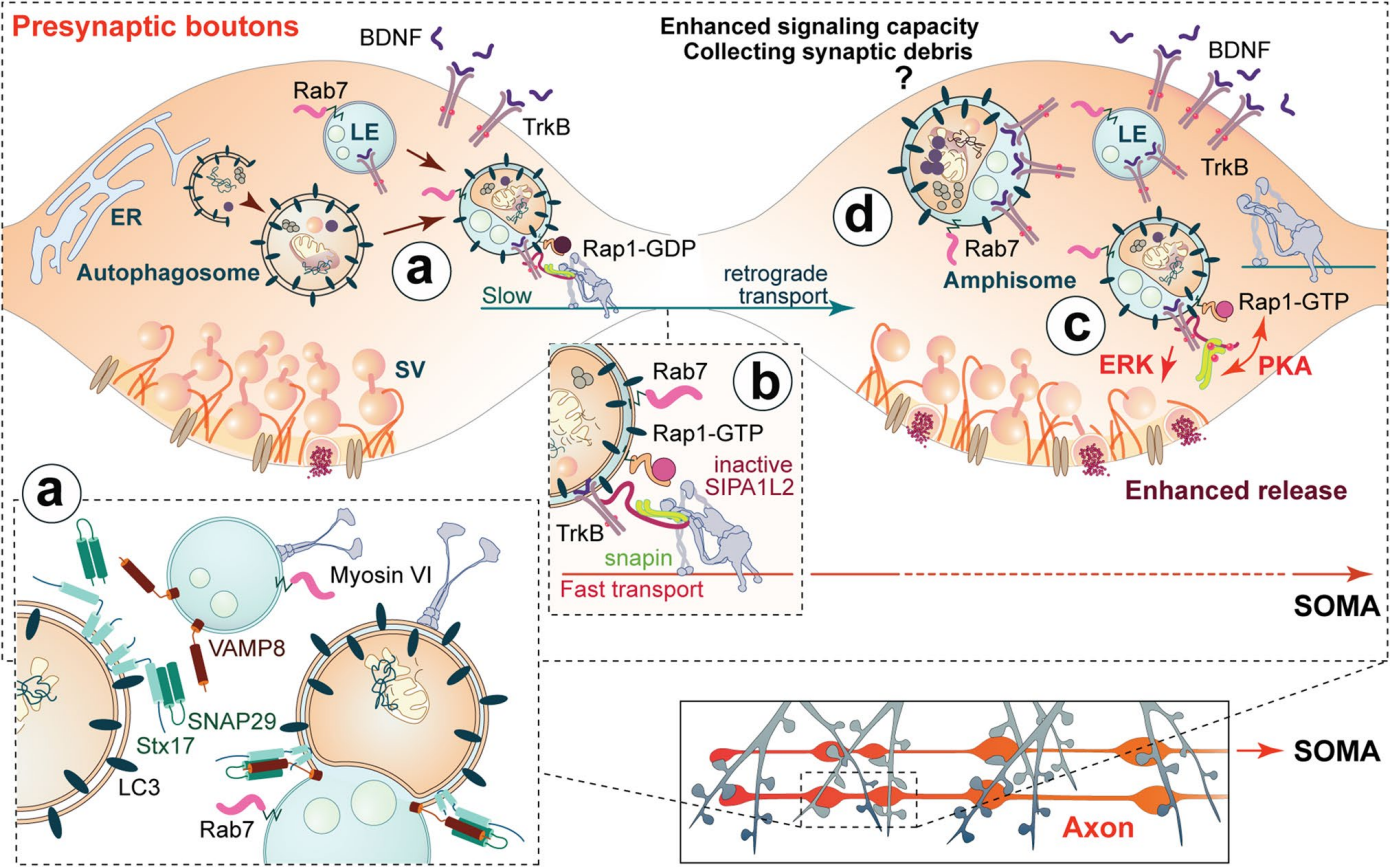
Local TrkB signalling at single boutons from TrkB-amphisomes containing SIPA1L2 and its potential implications for presynaptic plasticity. TrkB-amphisomes arise at presynaptic boutons by fusion of autophagosomes and late endosomes (**a**). Stx17/SNAP29/VAMP8 might control the fusion of both organelles. Myosin VI enable the delivery of endosomes to autophagosomes and its absence leads to an accumulation of immature autophagosomes and a reduced rate of protein aggregate clearance (**a**, inset). SIPA1L2 mediates the retrograde trafficking of TrkB-amphisomes by directly interacting with Snapin, the adaptor to dynein motors. RapGAP activity of SIPA1L2 is controlled by a direct interaction with the autophagy adaptor LC3b-II that in turns control both, the velocity (**b**) and signalling properties of the complex in a way that reduced RapGAP activity enables TrkB signalling. PKA activity promotes the stop of the complex at single boutons, reduces SIPA1L2 RapGAP activity that promotes local ERK activation and enhances neurotransmitter release (**c**). During these synaptic stopovers, TrkB-amphisomes might collect additional cargo, i.e., active TrkB receptors that would enhance the long-range signalling capabilities of the organelle (**d**). Trafficking of TrkB receptors in amphisomes would benefit from the double-membrane structure of autophagosomes, since TrkB receptors would be incorporated in the outer membrane and, therefore, separated from degradative cargo enclosed within the autophagosome inner membrane (**c**, **d**)

Transport from distal axons to the soma for cargo degradation is a common feature of both endosomes and autophagosomes and requires motor adaptors that connect cargoes to the dynein motor for retrograde trafficking. Multiple motor adaptors for the axonal transport of endosomes have been described and their mechanism of cargo interaction is generally well described [97–101]. Interestingly, the function of these motor adaptors is not restricted to the transport of endosomes but they are also essential for the transport of autophagosomes and autophagy-derived vesicles [100, 102–107], suggesting a common mechanism for retrograde transport of both organelles. How autophagosomes acquire these motors is not well understood and available evidence indicate that autophagosomes fuse to LEs to recruit dynein for retrograde transport (Fig. 3) [32, 104]. Fusion of  LEs with autophagosomes gives rise to hybrid organelles called amphisomes that in non-neuronal cells have a transient nature and rapidly transform into autolysosomes for degradation (see also Fig. 1). In neurons, however, they are likely to be continuously generated at distal axons and mature along their transport to the soma by incorporation of more endosomal cargo until their fusion with lysosomes (Fig. 3). In agreement with this, late endosomal compartments are connected to dynein motors via the motor adaptor Snapin (see above), a protein involved in the retrograde transport of endosomes [100], vesicles of the autophagy pathway [104] and TrkB-amphisomes [102].

Knowledge about biogenesis and function of amphisomes stems largely from studies performed in non-neuronal cells [108]. Syntaxin 17 (Stx17) is an autophagosomal Q-type *N*-ethylmaleimide-sensitive factor attachment protein receptor (Q-SNARE) that mediates fusion of this compartment with endosomes/lysosomes [109]. Stx17, which contains an LC3-interacting motif (LIR) is recruited to mature autophagosomes and localizes exclusively in their outer membrane [109]. This protein is able to recruit SNAP29 and the Stx17/SNAP29 complex binds to VAMP8, the R-SNARE interacting to Stx17 localized to endosomal and lysosomal membranes (Fig. 3a) [109]. Stx17 is so far the only known protein that mediates amphisome biogenesis in neurons, since shRNA-based reduction of Stx17 levels in primary neurons significantly reduced the number of amphisomes identified as LC3b-II and Rab7 positive structures [32]. This correlated with an elevated number of autophagic vacuoles lacking electron-dense cargo indicating a deficit in the maturation state of the organelle as a result of the impaired fusion between autophagosomes and late endosomes [32].

VAMP-3/Cellubrevin is another R-SNARE proposed to mediate the fusion of autophagosomes with MVB/LE in a cell line from human blood [110]. This protein is highly abundant in brain and has been shown to play a role in membrane trafficking in non-neuronal cells [111] but whether a similar role is found in neurons is currently unknown. Finally, a protein that might also be involved in amphisome biogenesis is Myosin VI, a motor protein with the unique property that it moves in the opposite direction as compared to other myosins. It plays a role in sorting of cargo in the endocytic pathway and it has also been shown to facilitate the delivery of endocytic cargo to autophagosomes in non-polarized cells via an interaction with Tom1, an alternative protein in the ESCRT-0 [112]. Myosin VI localizes to autophagosomes but also associates to Tom1 in endocytic compartments, thereby enabling the delivery of endosomes to autophagosomes (Fig. 3a). Absence of Myosin VI or Tom1 leads to an accumulation of immature autophagosomes and a reduced rate of protein aggregate clearance. Interestingly, targeting of Myosin VI to autophagosomes is mediated by autophagy adaptors [112], which might have implications for the selective incorporation of cargo to autophagosomes.

### Neurotrophin signalling at amphisomes: implications for neuronal plasticity

Neurotrophins are a family of soluble growth factors including the nerve growth factor (NFG) and the brain derived neurotrophic factor (BDNF) with an important role in the retrograde control of essential neuronal functions such as synaptogenesis, axonal growth and synaptic plasticity [113]. Signalling of these factors is initiated by binding to and activation of their corresponding tyrosine receptor kinase (Trk) receptor. The ligand–receptor complex is then internalized into signalling-competent compartments called signalling endosomes that are retrogradely transported causing the retrograde propagation of the signal [113]. Activation of Trk receptors initiates the activation of three molecular cascades, the PI3 kinase-, phospholipase Cγ- and Ras/MAPK-mediated pathways [113]. Isolation of retrograde carriers revealed the presence of activated Trk receptors and downstream molecular machinery from those pathways, as well as dynein and the dynactin complex [114]. Besides the association of these signalling compartments with the endosomal markers Rab5 and Rab7, the cellular identity of these endosomes is not currently clear. Several groups have recently looked into the crosstalk between signalling endosomes and autophagosomes. The use of inactive forms of presynaptically-endocytosed neurotoxins that are known to undergo retrograde transport in different populations of signalling endosomes has allowed the labelling and subsequent study of these compartments. Along these lines, the use of Botulinum neurotoxin (BoNT) which travels in signalling endosomes containing the neurotrophin receptor p75^NTR^ revealed that the number and retrograde flux of these signalling endosomes as well as autophagic vacuoles is dependent on presynaptic activity [47, 115]. Further ultrastructural analysis revealed that autophagosomes contained BoNT as cargo, thereby mediating the retrograde transport of this particular neurotoxin [47]. Importantly, this study suggests that cargo of autophagosomes is at least partially provided by the endolysosomal pathway in axons and it evidences that sorting mechanisms of cargo between the endolysosomal pathway and autophagy at boutons must exist to sort the toxin upon endocytosis to autophagosomes [47]. Nevertheless, this intersect between signalling endosomes and autophagosomes may only apply to a certain population of signalling endosomes. In fact, a subsequent study using Cholera Toxin subunit B (CTB), which partially overlaps to TrkB-positive signalling endosomes, showed that the cross-talk between these organelles and autophagosomes might be limited. This was suggested by experiments in which synaptic activation triggered an increased retrograde flux of TrkB signalling endosomes that was dependent on TrkB activation. Unlike the previous study, this was not the case for autophagosomes whose retrograde flux triggered by synaptic activity was not affected by TrkB inhibition, indicating that both organelles follow different routes [115]. However, since only a third of the TrkB population in axons overlapped with CTB, it is likely that different cellular pathways exist for the transport and signalling of TrkB receptors that might involve different organelles. In fact, recent evidence suggests that the transport of BDNF/TrkB receptors occurs in amphisomes to which the incorporation of activated TrkB receptors from LEs confers signalling capabilities (Fig. 3a) [102, 103]. In support of this, Kononenko and colleagues showed that TrkB receptors co-localize with LC3b-II-positive organelles that undergo retrograde transport relying on the dynein activator p150^Glued^ and the endocytic adaptor AP2 [103], as indicated by the accumulation of the receptor in immobile autophagosomes in the absence of AP2. Moreover, interruption of AP2-mediated transport induces an impairment of BDNF/TrkB signalling that is evident by a severe deficit in neuronal arborization in vivo and a decrease of BDNF expression levels, a major target of TrkB itself [103]. Importantly, conditional deletion of *atg5* in hippocampal neurons that prevents autophagosome biogenesis mimicked the deficits in neuronal arborization, suggesting that long-range transport of TrkB receptors in autophagosomes is essential for BDNF/TrkB-dependent regulation of gene expression [103]. TrkB-amphisomes do not only play a role in long-range BDNF/TrkB signalling but control TrkB signalling at single presynaptic terminals during the retrograde transport of the complex (Fig. 3) [102]. This temporal and spatial control of TrkB signalling is orchestrated by SIPA1L2, a RapGAP protein that serves as a motor adaptor of TrkB-amphisomes to a dynein motor by directly interacting with both, TrkB and Snapin. The RapGAP activity of SIPA1L2 inactivates Rap1, a protein necessary for the long-range TrkB-dependent activation of ERK1/2. In this molecular interplay, the autophagy adaptor LC3b-II regulates RapGAP activity of SIPA1L2 in a way that when LC3b-II is bound to SIPA1L2, TrkB signalling is inactivated. Unbinding of LC3b-II from SIPA1L2 downregulates its RapGAP activity and promotes the faster movement of the complex towards the soma (Fig. 3b) [102]. Stopover of the complex at single terminals is controlled by protein kinase A (PKA)-dependent phosphorylation of Snapin that induces the dissociation of TrkB-amphisomes from the dynein motor. PKA phosphorylation of SIPA1L2 also downregulates its RapGAP activity and allows local activation of ERK1/2 signalling at boutons and promotes neurotransmitter release (Fig. 3c) [102]. Impairment of this mechanism by gene deletion of *sipa1l2* has functional consequences in form of deficient presynaptic long-term plasticity at mossy fiber terminals and impaired spatial pattern separation [102], suggesting that this mechanism is relevant for presynaptic plasticity.

What would be the advantages of signalling from TrkB-amphisomes? Though ultrastructural evidence of neuronal amphisomes is lacking, characterization of amphisomes from rat liver tissue reveals them as organelles that resemble autophagosomes in having unaltered or slightly altered cytoplasm and that contain multiple, membrane-isolated “packages” that result from multiple fusion with endosomes and that altogether are surrounded by an outer membrane [116]. A similar structure of neuronal amphisomes would, therefore, allow the localization of activated TrkB receptors in the outer membrane, while degradative cargo would be contained within these “packages” or ILVs (see Figs. 1 and 3). In this way, activated TrkB receptors would benefit from the amphisome ultrastructure as it would physically separate them from luminal compartments with milder pH. Even though colocalization between amphisomes and pH-sensitive probes such as Lysotracker and LC3-GFP-RFP have been described in proximal regions of the axon [28], pH values revealed by these probes are far away from the optimal pH required by most lysosomal enzymes (~ 4.5, see [117]) and it is expected that, under these conditions, the organelle ultrastructure will be maintained without affecting the stability and signaling properties of TrkB receptors located in the outer membrane.

All in all, trafficking of TrkB receptors in amphisomes may ensure the long-range signalling capabilities of the receptor as lysosomes containing degradative enzymes are virtually absent in distal axons, and autophagosomes will need to reach proximal regions to acquire the degradative properties of autolysosomes. Of note, some colocalization between LC3-positive structures and reporters of catalytically active lysosomal enzymes has also been described [118], but this is found in developing neurons in which a role for lysosome-derived membranes in presynaptic assembly has also been reported [119]. Finally, this arrangement also opens up the possibility of new cargo acquisition in the form of signalling receptors which would take place by fusion events with receptor-bearing endosomes at a single bouton visited by the amphisome while undergoing retrograde trafficking. This would endow individual TrkB-amphisomes with different signalling capacities (Fig. 3d) that would be encoded in the amount of signalling receptors as well as in their diversity as cargo, as this may not only be limited to TrkB-receptors but include other signalling receptors. Overall, this could better integrate local synaptic function into long-range signalling. Recently, a study has revealed that BDNF acts as a negative regulator of autophagy in the forebrain and that inhibition of autophagy in hippocampus is required for induction of BDNF-dependent plasticity in vivo in some brain areas, revealing another link between TrkB signalling and autophagy [77]. How BDNF-dependent inhibition of autophagy affects long-range signalling from TrkB-amphisomes requires further study but one could speculate that upon inhibition of autophagy, newly-endocytosed BDNF/TrkB complex would get incorporated into already-formed TrkB-amphisomes rather than forming autophagosomes and this would potentiate the long-range signalling capacity of these organelles (Fig. 3d).

Finally, new evidence over the last years has uncovered different processes in which LC3b-II lipidation occurs in an autophagy-independent manner to single membrane vacuoles that ultimately fuse to lysosomes for cargo degradation [120–122]. This phenomenon has been termed as “non-canonical autophagy” and it has been reported to take place in pinocytosis, phagocytosis, pathogen invasion and apoptosis-independent cell death, though no evidence in neurons is reported so far [121]. Although transport of BDNF/TrkB complexes in endosomes carrying lipidated LC3b-II would likely not prevent the exposure of signalling receptor to degradative cargo in detriment of the receptor stability and its long-range signalling properties, due to the fact that most investigations discussed here have based their outcome in the assumption that LC3b-II lipidation only occurs in autophagy, it is worth mentioning that further investigations including ultrastructural analysis of TrkB-amphisomes are needed to better understand the biogenesis of these organelles.

## Open questions and concluding remarks

Autophagy and the endolysosomal system participate in the selective removal of presynaptic proteins and their function will have direct implications for synaptic transmitter release. Despite the progress made in recent years, several important questions still remain and should be addressed in future studies.

Firstly, critical questions relate to the cargo selectivity of each pathway. The intricate molecular machinery of presynaptic terminals demands highly regulated mechanism that achieve protein turnover in a coordinated manner, and this might be achieved by the combined function of the both pathways. If so, what is the molecular identity and biochemical properties of autophagy and endolysosomal cargoes? Are distinct protein classes predominantly degraded by one system or is there an overlap of cargo between pathways? How is this changed by synaptic activity? Along these lines, knowledge is sparse on the mechanisms underlying the turnover of presynaptic organelles such as SVs and ER. For instance, due to the implications of ER in calcium buffering at presynapses, could induction of ER-phagy serve as a mechanism to control ER size and thereby regulate neurotransmitter release?

Secondly, demands for autophagy vary among cell types and these demands might come along with different functional requirements for each neuronal cell type and might arise as a result of their divergent morphology. What are the mechanisms controlling autophagy induction in different cell types? Is autophagy differentially induced in axons and dendrites? Could these mechanisms be selectively induced at presynaptic terminals?

Thirdly, several contradictions in the field have to be resolved. For instance, why is the phenotype in autophagy ko mice relatively mild if a key process for SV integrity and protein aging are affected? Our rather fragmented knowledge about endosomal sorting processes at boutons currently makes an answer to this question difficult but, is it possible that, to a certain degree, endolysosomal and autophagic degradation substitute for each other?

Finally, several lines of evidence suggest that autophagy in neurons might serve synaptic plasticity processes and this might result from the intersection of both, autophagy and the endolysosomal system. How does biogenesis of signalling amphisomes occur and what is the cargo of these organelles? Since autophagy is necessary for memory formation and age-dependent cognitive decline is associated with reduced autophagy [6, 123], how will the reduction of autophagy affect signalling from TrkB receptors? And could the absence of TrkB signalling from signalling amphisomes also contribute to the decline in learning and memory processes observed with age? Recent years have seen a rapid development in this field and it is now timely to address these questions.
